# Relaxation training for anxiety: a ten-years systematic review with meta-analysis

**DOI:** 10.1186/1471-244X-8-41

**Published:** 2008-06-02

**Authors:** Gian Mauro Manzoni, Francesco Pagnini, Gianluca Castelnuovo, Enrico Molinari

**Affiliations:** 1 Istituto Auxologico Italiano IRCCS, Psychology Research Laboratory, San Giuseppe Hospital, Verbania, Italy; 2 Department of Psychology, University of Bergamo, Italy; 3 Department of Psychology, Catholic University of Milan, Italy

## Abstract

**Background:**

Relaxation training is a common treatment for anxiety problems. Lacking is a recent quantitative meta-analysis that enhances understanding of the variability and clinical significance of anxiety reduction outcomes after relaxation treatment.

**Methods:**

All studies (1997–2007), both RCT, observational and without control group, evaluating the efficacy of relaxation training (Jacobson's progressive relaxation, autogenic training, applied relaxation and meditation) for anxiety problems and disorders were identified by comprehensive electronic searches with Pubmed, Psychinfo and Cochrane Registers, by checking references of relevant studies and of other reviews. Our primary outcome was anxiety measured with psychometric questionnaires. Meta-analysis was undertaken synthesizing the data from all trials, distinguishing within and between effect sizes.

**Results:**

27 studies qualified for the inclusion in the meta-analysis. As hypothesized, relaxation training showed a medium-large effect size in the treatment of anxiety. Cohen's d was .57 (95% CI: .52 to .68) in the within analysis and .51 (95% CI: .46 to .634) in the between group analysis. Efficacy was higher for meditation, among volunteers and for longer treatments. Implications and limitations are discussed.

**Conclusion:**

The results show consistent and significant efficacy of relaxation training in reducing anxiety. This meta-analysis extends the existing literature through facilitation of a better understanding of the variability and clinical significance of anxiety improvement subsequent to relaxation training.

## Background

In recent years, it has been increasingly acknowledged that anxiety disorders are highly prevalent in the general adult population. Recent worldwide estimates for the 1-year and lifetime prevalence of any anxiety disorders are 10.6% and 16.6%, respectively, with a ratio indicating that a large number of people experience anxiety disorders on a continuing or recurring basis. Prevalence is approximately twice among women, with overall age-specific rates remaining relatively stable or increasing across the lifespan [1].

Moreover, anxiety disorders constitute only the tail of the curve representing the general anxiety distress that affects the population. According to Zigmond and Snaith [2], psychiatric disorder cannot be considered either present or absent since the degrees is continuously distributed in the population. In fact, complaints of anxiety are common among healthy individuals and have been associated with numerous negative health consequences [3,4], absenteeism and decreased work productivity [5]. Studies have persistently shown that anxiety disorders produce morbidity, utilization of health care services, sometimes for long time, functional impairment [6] and personal distress, leading to a burden of both private and public health care costs.

The prevalence of anxiety disorders, both in their severe and mild forms, is certainly high also in medical and surgical departments [6,2]. Emotional distress presented by in- and out-patients may be a result of the stress caused by physical illness and, more subtle, somatic symptoms presented may be a manifestation of anxiety states, with no basis in organic pathology [7].

A broad understanding of the etiology of anxiety problems includes a multiplicity of factors, such as biological, psychological, and social determinants, which are mediated by a range of risk and protective factors [1]. The old debate over the primacy of these factors, overall biological or psychological, is gradually being replaced by a pragmatic model considering all the relative contributions [8].

Clinical trials have shown that anxiolytic drugs alone have limited long-term efficacy [9]. Moreover, they often have adverse side effects including dependency, drowsiness [10], impaired cognition and memory [10,11] and sexual dysfunction [11-13]. Consequently, clinical community has begun to consider alternative old and new approaches targeting anxiety problems and to examine the merits of combined and tailored somatic and psychological treatments.

Huge progress has been made (and still goes on) in the nonpharmacological treatment of anxiety disorders [14]. In this direction, relaxation techniques represent one of the most used approach in anxiety management worldwide, both as a stand-alone treatment or included in a more complex therapy.

Even if there are many relaxing methods that have received scientific attention, they could be defined globally as a cognitive and/or behavioral treatment approach which emphasizes the development of a relaxation response to counteract the stress response of anxiety. The relaxation response is defined by a set of integrated physiological mechanisms and 'adjustments' that are elicited when a subject engages in a repetitive mental or physical activity and passively ignores distracting thoughts [15].

Many studies support a good efficacy of relaxation trainings in reducing anxiety. For example, in a study by Kanji, White and Ernst [16], fifty-nine patients were randomly assigned to receive regular autogenic training or no such therapy as an adjunct to standard care for 5 months. State Anxiety showed a significant intergroup difference both at 2 and 5 months. This finding was corroborated by secondary outcome measures, for example quality of life, and by qualitative information about patients' experiences, suggesting that autogenic training may have a role in reducing anxiety of patients undergoing coronary angioplasty.

Moreover, in a general review on therapeutic use of relaxation response in stress-related diseases, Esch et al. [15] declare that relaxation techniques appear to be highly recommendable. Many studies have been conducted that have shown a positive clinical outcome of the relaxation techniques in connection with anxiety [17-26]. A review conducted by Kanji and Ernst [27], considering 8 studies, suggests that autogenic training seems to reduce stress and anxiety, but few conclusion can be drawn from those studies. Carlson and Hoyle [28] wrote a quantitative review focused on progressive relaxation training [29], indicating a good potential of progressive relaxation in the treatment of various diseases (i.e. migraine, hypertension, chemotherapy side effects...) but without specific consideration about anxiety.

An old meta-analysis [30], published in 1989 about the effects of relaxation trainings on trait anxiety found that relaxation techniques had a medium effect size, while transcendental meditation had significantly larger effect size.

Applied Relaxation has been adopted for uses in treatment of generalized anxiety disorder [31]. In two recent studies, applied relaxation has proven to be equally as effective in treating GAD as Cognitive therapy, which demands much more of the therapist [31,32].

Though there is much research which has combined meditation therapy with conventional treatment in anxiety disorders, there is still a lack of reviews that provide substantial evidence on the effectiveness of meditation therapy programs, both for short-term and long-term effects and for acceptability in terms of practicality, feasibility, difficulty and concerns about the adverse effects.

Meditation is sometimes considered to be a form of relaxation therapy, however meditation not only creates a relaxation response but also produces an altered state of consciousness which facilitates the meta-cognitive mode of thinking which make possible the expectation of cognitive-behavioral benefits. Meditation is effective against anxiety, both if considered as a single treatment [8,33] or inserted into a cognitive therapy. For example, Finucane and Mercer [34] applied Mindfulness Based Cognitive Therapy (MBCT) in an 8-week course that integrates mindfulness meditation practices and cognitive theory to patients with recurrent depression or recurrent depression and anxiety, finding a great average reduction of anxiety, as well as depression.

The aim of this meta-analytic study was to investigate the efficacy of relaxation training programs which are currently used to treat anxiety disorders and to reduce anxiety in general. This idea derived from a need we had in our clinical practice, to collect some information about the relaxation methods recently most used in clinical trials, both randomized or observational, and about their relative efficacy in reducing anxiety in different samples. During the preliminary search of the literature, we decided to organize a review of the studies published in the last ten years. We chose this span of time because we thought that ten years are an appropriate timeframe to make a picture of the current situation in the field of relaxation techniques for anxiety management. Further, we thought that in ten years there would have been enough studies to allow meta-analytical calculations.

This study employed a meta-analytic approach to test several hypotheses derived from the extant literature. Hypothesis 1: Post-treatment anxiety would be lower than baseline level, and relaxation training would outperform control conditions (where presents) on anxiety-specific measures. Hypothesis 2: there would be significant differences between the different relaxing approaches considered. Hypothesis 3: there would be a difference in anxiety reduction between subjects with physical and psychological diseases. Hypothesis 4: there would be a dose-response relationship for relaxation training. Hypothesis 5: the suggestion of practicing relaxation exercises at home would enhance the efficacy of the training. Hypothesis 6: the context of application (individual or group sessions) would moderate the outcome. Hypothesis 7: Different anxiety questionnaires would present different sensitivity to anxiety changing.

## Methods

### Study selection

The overall objective of study selection was to collect published journal articles that examined anxiety level before and after relaxation training for reduction of anxiety both in clinical and non-clinical population.

We searched the following databases: PsycINFO, MEDLINE and the Cochraine Central Register of Controlled Trials. The searches were restricted to the past ten years (1997–2007) and included the following terms: relaxation training, relaxation exercise(s), relaxation therapy, autogenic training, relaxation AND meditation, relaxation. These words were searched as key words, title, abstract, and MeSH subject heading terms. Also, citation maps were examined and the "cited by" search tools was used. These findings were cross referenced with references from reviews. Findings were limited to human adults and English language studies. We didn't consider unpublished works.

### Study elegibility

Two reviewers (GMM and FP) screened the abstracts of all publications obtained by the search strategy. Studies meeting the following inclusion criteria were selected for the meta-analysis: (a) at least one relaxation training condition (no matter if it was the object of the paper or the treatment of the control group), (b) reporting of interval or ratio data, (c) use of psychometrical questionnaires; (d) anxiety level data presented before and after relaxation training, (e) sufficient reporting of study results (e.g. means and standard deviations) to allow for effect size computation. It is important to note that some studies were both repeated measure designs (before and after relaxation training), as well as comparisons (relaxation training versus control or other conditions). A distinction was not made among studies in which relaxation training alone or in combination was compared to a comparison/control group, studies in which relaxation training was examined without a control element and observational trials. The absence of a control group was not an exclusion criterion, because effect size's calculation can be done also on pre-post modifications. However, since between group and within group analyses are methodologically different, two separated analyses were conducted.

### Data coding

For all papers selected, the full articles were obtained and inspected to assess their relevance, based on the preplanned criteria for inclusion. Data were independently extracted by two reviewers (FP and GM) using a predesigned data collection form: (1) number of subjects; geographic origin of the study; relaxation training type; (4) subjects typology; (5) mean age and women percentage; (6) assessment measures; (7) homework; (8) protocol length; (9) trial context; (10) summary statistics required for computation of effect sizes. Any disagreements were discussed with a third reviewer (GC).

### Study characteristics

Besides computing the total average effect size relaxation training has on anxiety (separately for controlled and non-controlled studies), also the specific average effect sizes related to the different approaches considered were computed. The relaxation methods included are: autogenic training, Jacobson progressive relaxation, meditation and Benson's technique (considered together, given their similarities), applied relaxation, a combination of two or more methods (i.e. autogenic training in combination with visualization), other techniques.

In controlled studies, comparison condition consists in waitlist, simply laying down on a relaxing chair or on a bed, non-specific relaxing activities (i.e. reading a newspaper).

Specific average effect sizes were calculated also for type of subjects, who have been divided into three large groups, since the sample wasn't broad enough to conduct an higher diagnostic differentiation. The first category is represented by volunteers (i.e. workers) or students (high school or academic). The second one is composed by patients with medical diseases (i.e. irritable bowel syndrome). The last one represents patients with psychological or psychosomatic disorders. Participants have been inserted as psychosomatics patients only if well specified.

A number of moderators were considered: age, gender, context of training (individual or group), duration (expressed in days), use of homework (repetition of relaxation exercises with or without audiotapes), psychometric questionnaire used and studies geographical provenience.

When age was not reported, it was estimated on the base of other data (i.e. the year of school). Ambiguity concerning studies sample sizes (i.e. unspecified attrition) was solved with the conservative approach of using the smallest number for which there was clear documentation.

Anxiety is the dependent variable and only subjective assessments were considered. From the studies selected for the meta-analysis, we decided to extract psychometric data mainly from three questionnaires: the Spielberger's STAI (State-Trait Anxiety Inventory) [35], the HADS (Hospital Anxiety and Depression Scale) [2,36] and the BAI (Beck Anxiety Inventory) [37]. Considered studies used also other questionnaires assessing anxiety, but we chose to exclude them from the categorization because of their paucity in our sample of studies.

### Calculation of the Effect Sizes

Effect sizes were calculated for all studies, both within and between group (where possible). This means that some researches contribute to both the between groups and the within group meta-analyses.

Between groups effect sizes for studies with control or comparison group were computed using Cohen's d [38,39]. When the necessary data were available, all effect sizes were calculated directly using the following formula: d = (M1 – M2)/S, where M1 is the mean of the treatment group, M2 the mean of the comparison group and S is the standard deviation for the pooled sample, calculated with the following formula: √ (n1-1) s12+(n2-1)s22)/n1+n2-2, where n1 is the number of subjects in the experimental group, n2 that of the control group and s is the standard deviation of groups.

For within group studies without control group, baseline scores have been used instead of control group in the above formulas.

If these data were not provided, d was estimated using conversion equations for significance tests [40]. These effect sizes may then be interpreted with Cohen's convention [38] of small (0.2), medium (0.5) and large (0.8) effects. The overall mean effect size for all of the studies combined was weighted by the variance of the studies, considering both standard deviations and subjects number.

Prior to combining studies in the meta-analysis, we assessed the homogeneity of the effect sizes [39]. Cochran's Q-statistic [41] was computed by summing the squared deviations of each study's estimate from the overall meta-analytic estimate, weighting each study's contribution in the same manner as in the meta-analysis [39].

The fail-safe N [42] for ES was also calculated. This is a hypothetical estimator dealing with the problem of an incomplete retrieval of studies. The fail-safe N demonstrates how many file-drawer studies with an assumed ES of zero are necessary to reduce the ES of the meta-analysis to a given level.

## Results

### Between groups analysis

We located 19 studies (see table 1) with a random allocation of subjects into a relaxation training treatment or in a control/comparison group. The pooled sample was composed by 1005 subjects, whose 568 were allocated in the experimental training groups, while 437 were included in control/comparison groups. The mean age is 33,27 years, mostly women (62,75%). In 8 studies (42%), the sample is composed by people with physical diseases, in 6 (31,6%) by volunteers or students, in 5 (26,3%) by psychological or psychosomatic patients. Progressive relaxation was used in 10 works (41,7%), autogenic training and meditation in 2 (8,3%). Only one study used applied relaxation. The other researches evaluated the effects of multi-methods training (3 studies, 12,5%) or other techniques (6, 25%). Half the papers were North American publications (9, 47,4%), 5 were Asian (26,3%), 3 European (15,8%) and 2 Oceanian (10,5%). The most used instrument was the state form of the STAI (14 studies, 73,7%). Only one study used the trait Scale, so as the Beck Anxiety Inventory. Two works assessed the level of anxiety with the Hospital Anxiety and Depression Scale. The context of training was equally divided between individual (52,6%) and group sessions (47,4%). The most part (68,4%) of the trainings required (or, at least, recommended) implementing some activities at home or outside the clinical setting.

**Table 1:** Characteristics of the studies.

**Study**	**N° Subjects**	**N° Controls**	**Mean Age**	**% Women**	**Country**	**Type of training**	**Type of Subjects**	**Instrument**	**Individual/group**	**Homework**	**Duration**	**Session**	**Effect size BT**	**Effect size WT**
* Kanji, White,& Ernst [16]	30	29	64,5	36,7	UK	Autogenic Training	Patients undergoing coronary angioplasty	Stai – State anxiety	Group	Yes	2 Months	ND	0,470	0,513
* Tomioka & Kubo [50]	21	12	43,3	72,7	Japan	Autogenic Training	Psychosomatic patients	Stai – State anxiety	Group	Yes	8 Weeks	8	0,318	0,399
* Pawlow, O'Neil & Malcolm [21]	10	10	38	90	USA	Progressive Relaxation	Night Eaters	Stai – State anxiety	Group	Yes	1 Day	1	1,358	1,305
* Yung, Fung, Chan, & Lau [24]	17	30	37	100	Hong Kong	Stretch Release Relaxation Group;	Nurses	Stai – State anxiety	Group	Yes	4 Weeks	4	0,397	0,210
* Yung, Fung, Chan, & Lau [24]	18					Cognitive Relaxation					4 Weeks	4	0,623	0,331
*Roykulcharoen & Good [23]	51	51	42	82,3	Thailand	Systematic Relaxation	Patients underwent abdominal surgery	Stai – State anxiety	Individual	No	1 Day	1	0,176	0,371
* McComb & Clopton [51]	26	24	19	100	USA	Autogenic Training and Visualization	Pazients affected by bulimia nervosa	Stai – State anxiety	Group	No	8 Weeks	8	0,030	0,262
* Deckro et al. [52]	46	44	24	60,2		Relaxation response and other cognitive-behavioral techniques	Students	Stai – State anxiety	Group	Yes	6 Weeks	6	0,700	0,575
* Pawlow & Jones [20]	44	15	23,2	47,8	USA	Progressive Relaxation	Students	Stai – State anxiety	Individual	No	1 Day	1	1,389	1,054
* Knowlton & Larkin [53]	12	12	20,8	100	USA	Progressive Relaxation, recommended voice	"Very anxious" students	Stai – State anxiety	Individual	No	1 Day	1	0,160	0,731
* Knowlton & Larkin [53]	12					Progressive Relaxation, conversational voice					1 Day	1	0,537	1,491
* Knowlton & Larkin [53]	12					Systematic Relaxation					1 Day	1	0,497	0,929
Wachholtz & Pargament [54]	22		19,40	63,6	USA	Unspecified Relaxation	Students	Stai – Trait anxiety	Individual	Yes	2 Weeks	2		0,188
Wachholtz & Pargament [54]	25		18,90	76		Spiritual Meditation					2 Weeks	2		0,679
Wachholtz & Pargament [54]	21		19,10	61,9		Secular Meditation					2 Weeks	2		0,211
* Bagheri-Nesami, Mohseni-Bandpei, & Azar [55]	26	24	48	96	Iran	Benson's Technique	Rheumatoid arthritis patients	Stai – State anxiety	Individual	Yes	8 Weeks	1	0,950	1,200
* Rasid &Parish [56]	18	17	18	50	USA	Behavioral Relaxation	High school students	Stai – State anxiety	Group	Yes	2 Weeks	4	0,900	
* Rasid &Parish [56]	20		18	55		Progressive Relaxation		Stai – State anxiety	Group	Yes	2 Weeks	4	0,900	
Wright, Courtney, & Crowther [57]	18		40	ND	Irland	Autogenic Training	Patients with cancer	Hospital Anxiety and Depression scale – HADS;	Group	Yes	10 Weeks	10		1,350
* Boyce, Talley, Balaam, Koloski, & Truman [58]	19	25	42,3	81	Australia	Progressive Relaxation	Patients with Irritable Bowel Syndrome	Hospital Anxiety and Depression scale – HADS;	Individual	Yes	4 Weeks	4	0,096	0,411
* Norton, Holm, & McSherry [59]	17	16	20,3	70	USA	Progressive Relaxation	Students	State-Trait Personality Inventory	Individual	Yes	2 Weeks	2	0,265	1,138
Carlbring, Ekselius, & Andersson [60]	11		37,4	63	Sweden	Applied relaxation	Pazients with panic	Beck Anxiety Inventory	Individual	Yes	7 Months	9		0,950
Muhlberger, Herrmann, Wiedemann, Ellgring [61]	15		42,2	86,7	Germany	Progressive Relaxation after the exposure of Virtual Reality	Flight phobics	Anxiety Expectancy Scale	Individual	No	1 Weeks	4		0,072
Viens, De Koninck, Mercier, St-Onge, & Lorrain [62]	10		36,1	80	Canada	Progressive Relaxation, management of anxiety	Subjects with sleep disorders	Stai – State anxiety	Individual	Yes	9 Weeks	1		0,865
* Davidson et al [63]	25	16	36	76	USA	Meditation	Employers	Stai – Trait anxiety	Group	Yes	8 Weeks	8	0,747	0,545
* Lukins, Davan, & Drummond [64]	43	52	47	50,8	Australia	Imaginative Relaxation	Patients undergoing magnetic resonance	Stai – State anxiety	Individual	No	1 Day	1	0,526	0,174
* Lukins, Davan, & Drummond [64]	44					Imaginative Relaxation with re-calling during MRI					1 Day	2	0,384	0,050
* Cheung, Molassiotis, & Chang [17]	8	10	48,8	37,5	Hong Kong	Progressive Relaxation	Patients after stoma surgery	Stai – State anxiety	Individual	Yes	1 Day	2	0,479	
Kominars [65]	76		nd	nd	USA	Progressive Relaxation and Visualization	Patients alchol addicted	Stai – State anxiety	Group	nd	3 Weeks	6		1,010
Arntz [31]	20		35,9	60	Netherland	Applied relaxation	Patients with Generalized Anxiety Disorder	Stai – Trait anxiety	Individual	Yes	12 Weeks	12		0,369
Engel & Andersen [66]	8		48,5	37,5	Denmark	Guided Relaxation and Meditative Streching	Patients with chronic toxic encephalopathy	Stai – State anxiety	Group	No	8 Weeks	8		0,549
* Jablon, Naliboff, Gilmore, & Rosenthal [67]	10	10	58,9	50	USA	Progressive Relaxation and EMG biofeedback	Patients with type II diabetes	Stai – State anxiety	Individual	Yes	4 Weeks	4	0,254	0,412
* Clarl et al. [68]	19	20	31,95	44	UK	Applied relaxation	Patients with social phobia	Beck Anxiety Inventory	Individual	Yes	14 Weeks	14	0,949	1,029
* Lowe et al. [69]	20	20	63,1	20	Germany	Progressive Relaxation	Patients After Acute Myocardial Infarction	Hospital Anxiety and Depression Scale	Group	No	1 Week	2	0,189	-0,061

* = Studies with control group

#### Overall efficacy of relaxation training

The average effect size, weighted by the pooled variance, is .5136 (95% CI: .46–.634). This result indicates a medium-high efficacy, according to Cohen's convention. The range of effect sizes is considerable (from .03 to 1.389), which contributes to a significant test of heterogeneity Q, χ (18) = 28,93, p < .05. This significant heterogeneity of effect sizes suggests that the overall efficacy of relaxation training must be handled with caution because of the differences among the relaxation approaches considered, the kind of subjects and the questionnaire used.

The fail-safe n (for k = 19 interventions and the overall mean d of .5136) tells us that we would need an additional 79 studies with non-significant findings in order to reduce the mean d to a small effect size (.1).

#### Effect sizes by relaxation training types

Applied relaxation shows an higher effect size in comparison with all other treatments (p < .01), but not with meditation. However, this result is not reliable because applied relaxation was used just by one study. Meditation proved to be very effective in the reduction of anxiety, statistically superior to the other techniques (p < .01 against progressive relaxation, autogenic training, multi-methods, other techniques). All the other techniques show good efficacy, even if statistically lower than meditation (table 2).

**Table 2:** Effect sizes in between analysis (BT)

**Variable**	**ES BT**
*Relaxation technique*	
Autogenic Training	,41791
Progressive Relaxation	,55404
Meditation	,85881
Applied relaxation	,94900
Multi-modality	,42650
Other techniques	,43118
	
*Typology of subjects*	
Students/Volunteers	,73034
Patients with physical diseases	,38598
Patients with psychological of psychosomatic diseases	,46727
	
*Group/Individual*	
Group	,55136
Individual	,48369
	
*Homework*	
Yes	,61482
No	,39472
	
*Assessment*	
STAI – State anxiety	,53128
STAI – Trait anxiety	,74700
Hospital Anxiety and Depression Scale	,14055
Beck Anxiety Inventory	,94900
Other questionnaires	,61879

#### Effect sizes by kind of subjects

Varying the type of subjects, the effects relaxation training has on anxiety change significantly. Volunteers and students show a reduction greater than other types (p < .001 in both cases). There were no differences between medical and psychological patients (table 2).

#### Moderator variables analysis

At study level there is a negative correlation between the average age of subjects and the effect sizes, indicating that young people gain more benefits. There is also a negative correlation between the percentages of women and effect sizes. However, women's presence is higher in studies with psychological and psychosomatic patients.

The context of implementation doesn't seem to influence significantly the efficacy of treatment, even if group sessions have an higher average score than the individual ones. At study level there is a positive correlation (p < .05) between the length of treatment and its effect size. Homework increases effect size in comparison to the therapist's sessions alone (p < .001). There are also differences of effect size among the instruments used for psychometric assessment. Studies that used the Hospital Anxiety and Depression Scale show lower results (p < .001) in comparison to the other questionnaires, which don't differ significantly from each other (table 3).

**Table 3:** Correlation between effect sizes and moderators (BT)

**Moderator**	**r with ES BT**	**P**
Mean age	-.237	<.001
Women presence	-.213	<.01
Duration of training	.186	<.01

### Within group analysis

This analysis is based on 25 studies (see table 1), with a total sample of 748 participants.

The mean age of the sample is 32,65 years, with a higher percentage of women (59,5%). In 10 studies (40%) the sample was composed by patients with psychological or psychosomatic diseases, 9 (36%) by patients with somatic troubles and 6 by volunteers or students.

Progressive relaxation is the most studied training among the papers included in this meta-analysis of observational studies (33,3%). Autogenic training, meditation and applied relaxation were implemented in 3 studies each (11,1%). In 4 papers (14,8%) a multi-methods training was implemented, while in others 5 (18,5%) other techniques.

The state scale of the STAI is the most administered questionnaire (15 studies, 60%). The trait scale was found in 3 papers (12%), as like the Hospital Anxiety and Depression Scale, while 2 papers (8%) used the Beck Anxiety Inventory. The context is mainly individual (14 studies, 56%) and homework is suggested in two third (66,7%) of the papers. A great part of the works come from USA or Canada (44%), a third from Europe (32%), 4 (16%) from Asia and 2 from Oceania (8%).

#### Overall efficacy of relaxation training

The average effect size is .57 (95% CI: .52–.68) and, according to Cohen's categories, is a medium-high score. The range of the results is quite wide (from -.061 to 1,49). Effect sizes are not homogeneous, χ (30) = 55.469, p < .01. This significant heterogeneity suggests that the overall effectiveness of relaxation training must be handled with caution because of the differences among the relaxation approaches considered, the kind of subjects and the questionnaire used, as it's for the between group analysis.

Failsafe N calculation indicates that an additional 118 studies with an effect size value of zero would be needed to reduce the effect size toward the value of 0.1.

#### Effect sizes by relaxation training types

Progressive relaxation, applied relaxation, autogenic training and meditation show great efficacy in decreasing anxiety against the combination of more than one methods and the other techniques. The "others techniques" treatment type shows the lowest score (table 4).

**Table 4:** Effect sizes in within analysis

**Variable**	**ES WT**
*Relaxation technique*	
Autogenic Training	,66747
Progressive Relaxation	,82439
Meditation	,66236
Applied relaxation	,73198
Multi-modality	,46241
Other techniques	,21614
	
*Typology of subjects*	
Students/Volunteers	,58370
Patients with physical diseases	,40239
Patients with psychological of psychosomatic diseases	,75459
	
*Group/Individual*	
Group	,62322
Individual	,58885
	
*Homework*	
Yes	,65462
No	,40640
	
*Assessment*	
STAI – State anxiety	,59195
STAI – Trait anxiety	,40826
Hospital Anxiety and Depression Scale	,49247
Beck Anxiety Inventory	,99956
Other questionnaires	,59871

#### Effect sizes by kind of subjects

Comparing values before and after the treatment (table 4), the category of subjects with psychological and psychosomatic diseases had higher decrease of anxiety level in comparison with volunteers (p < .01) and with participants with medical problems (p < .001). Subjects with medical problems show a less decrease of anxiety also in comparison to volunteers and students (p < .01).

#### Moderator variables analysis

At study level, the average age of the samples correlates negatively with the effect size, indicating that older people has a smaller reduction of anxiety in comparison with younger. A positive correlation emerges also between the percentage of women and the effect size. However, also in this analysis there is a higher presence of women in the studies with psychological and psychosomatic patients. The context of treatment doesn't seem to moderate the treatment effect. In fact, there are no significant differences between group and individual sessions. Larger effect size corresponds to a higher number of days on treatment (p < .001). The suggestion to apply relaxation techniques at home, together with relaxation sessions conducted by a therapist, increases the effect size of the treatment (p < .001). Effect sizes are really influenced also by the chosen assessment instrument. The questionnaire associated with the higher effect size is the BAI in comparison with the other scales (p < .01). State scale of the STAI-Y shows an higher effect size than the Trait Scale (p < .05). With the exception of the BAI, there wasn't any statistical difference between HADS and other instruments (table 5).

**Table 5:** Correlation between effect sizes of within analysis and moderators

**Moderator**	**r with ES WT**	**p**
Mean age	-.268	<.001
Women presence	.145	<.01
Duration of training	.243	<.001

## Discussion

The two analyses presented above primarily evaluated the impact that relaxation training has on anxiety in general. Certainly, there is a methodological difference between the two types of data. In the between groups analysis, effect sizes are computed from the difference between experimental and control group. Thus, it is possible to distinguish the effects produced by the relaxation treatment from those caused by the simple passing of time. This is not possible when the evaluation of the treatment depend on the differences between the score measured before and after the training. Any change observed would depend partially from treatment, from the simple passing of time and from others uncontrolled variables.

In any case, both meta-analyses indicate a good efficacy of relaxation training in the reduction of anxiety, both in comparison with a control group and with the participants as controls for themselves.

This result is aligned with the research literature [33,28,30,8,25,26] and with relaxation manuals indications [43-45].

There is a great heterogeneity of effect sizes. In order to reduce this variability, some distinctions have been made. All the relaxation techniques considered show a good potential in the reduction of anxiety. Applied relaxation and meditation have very high effect scores both in within and between analyses. However, in latter analysis, applied relaxation is used only in one study, making this result not valid. Progressive relaxation produced high effect sizes, with a within group reduction superior to the other techniques. The decrease in anxiety obtained with autogenic training is a little lower (but still positive) than other techniques in the between groups comparison, but its within group effect size is aligned with the general average. A multi-techniques approach does not increase relaxation training efficacy on anxiety reduction, showing an effect size level relatively low in both analysis. Non codified techniques, alias "other techniques", represent the category with the lowest score, especially in the within group analysis.

The selection of the best relaxation technique is quite hard. The high effect size levels reached by meditation, applied relaxation and progressive relaxation may indicate a good efficacy in the reduction of anxiety from all of them. An indication that seems to rise from those data is to apply just one model, avoiding the use of more techniques together.

There is a difference between the two analyses concerning the typology of participants. In between groups analysis, volunteers and students have an higher reduction of anxiety. Within group analysis indicates a good efficacy for this category, despite a lower score than the former one. Patients with psychological or psychosomatic diseases present different results between the two analyses. In controlled studies, the average effect size is medium-low, while open trials without control group indicate a really higher effect size. Globally, at baseline, participants with psychological or psychosomatic diseases show higher anxiety levels in comparison with the other and this can explain greater differences between pre and post assessment in within group studies. Control groups of studies with psychological diseases may help to understand the data of the between group analysis. In fact, there seems to be a waiting list effect [46], because people often improve just by being in a waiting list. Moreover, some people could have been under an unknown treatment (psychotherapy, pharmacological...) leading to an uncontrolled anxiety decreasing. Differently, people without a particular disease (students or volunteers) present a stable level of anxiety along time and treatment effect is "pure", because not related to an expected "physiological" decreasing of anxiety from higher levels in clinical samples.

An opposite correlation between effect size and percentage of women emerged between the two meta-analyses. This correlation is negative in the between group analysis, while it's positive in the within group one. This result is hard to explain. Maybe it is related to an heterogeneous percentage of women in the different groups of subjects. For example, in the samples with psychological or psychosomatic problems there is a significantly greater presence of women. So, this result may depend mostly on samples composition, and must be taken with caution. Further research is needed.

Patients with medical problems presented the lower effect size, both in within group and between group analyses, with medium-low efficacy. However, for this patients, the objective of relaxation is not the reduction of anxiety. More often relaxation techniques are used to reduce perceived pain or somatic symptoms (i.e. nausea, hypertension).

There is a negative correlation also between the efficacy of relaxation training and the mean age in both meta-analyses. Older people have less benefits than younger. Older people may have also more difficult in the practice of physical exercises (i.e. in the Jacobson's progressive relaxation training) or, maybe, a lesser understanding of instructions.

The context of application doesn't moderate the reduction of anxiety, in contrast to what found by Carlson and Hoyle [28], who indicate individual treatment as more effective (but their analysis was about the effects of progressive relaxation on various pathology).

The efficacy of the treatment increases with the duration of the protocol, in both meta-analyses. Repetitive training over a long period product significantly higher modification. Maybe there is an expected correlation between the amount of time spent in practicing exercises and their efficacy. In fact, effect size increases significantly with the request of practicing the exercises at home, consistent with past findings [47].

Finally, concerning the anxiety questionnaires, studies that used the Trait scale of the STAI show a lower within group effect size compared to those that used the State scale. This result is coherent with the different theoretical constructs measured by the STAI: changing a trait is harder than changing a state. In the between group meta-analysis, there was only one study that used the Trait scale, so no generalization can be done.

The State scale of the STAI is the most used instrument in the present sample of papers and the effect sizes are similar between the two meta-analysis.

The anxiety scale of HADS showed a low between groups effect size and a medium within group effect size. The interpretation of this data is quite complex, because, against the less discriminant result in the intergroup analysis, international literature demonstrated good psychometric properties of the instrument [48]. A possible explanation deal with the main target of the scale, that assessed usually hospital patients, with a severe physical problem (i.e. cancer).

The higher effect sizes come from the studies that used the BAI, but these are too few in order to make a generalization (one in the between analysis, two in the within one).

## Limitations

Findings from this meta-analysis must be interpreted with caution given limitations of meta-analysis in general and of data collected for this analysis in particular.

A critical issue for this meta-analysis, as is true of any systematic review, was deciding which trials or studies to include and which to exclude. While some researchers (e.g. Cochrane Collaboration) view the randomized trial (RCT) as the only acceptable evidence on treatment outcome, many systematic reviews are indeterminate because they include insufficient RCTs whilst they reject large numbers of non-randomized controlled studies.

We decided to include all studies published and relevant to our aim, independently from their research design, in order to increase the number of studies and participants. However, within group meta-analysis we conducted is very limited because it is impossible to state if anxiety enhancements were directly related to or caused by relaxation training.

As in any review of studies in a given area, it is possible that studies with non significant results are underreported. The practice of publishing only studies with significant outcomes may create a distortion of the subject under investigation, especially if a meta-analysis is done [49].

It is important to note that, for some variables, meta-analyses were based on relatively few subjects.

We searched studies in the most important databases for psychology (PsychInfo) and medicine (Medline). Other databases (e.g. CINAHL) were not screened and this may be a limitation to the generalizability of our results.

## Conclusion

Notwithstanding its limitations, the present meta-analytical study show consistent and significant efficacy of relaxation training in reducing anxiety, coherently with past studies and reviews [26,30,28,27]. The first hypothesis is then confirmed: post-treatment anxiety is lower than baseline level and relaxation training outperforms control conditions on anxiety-specific measures.

While all relaxation trainings reduced anxiety, applied relaxation, progressive relaxation and meditation showed greater effect sizes than other techniques. In particular, this meta-analysis evidences the lower potential of multi-methods relaxation. The use of one of the main relaxation techniques is preferable, at least for anxiety reduction.

Both psychological or psychosomatic patients and volunteer subjects gain more benefits from relaxation training. The reduction of anxiety for medical patients is lower in comparison to the others categories, but relaxation training still has good efficacy.

It is possible, even if it should be investigated by further studies, that young people can have a better decreasing of anxiety levels, compared to old people.

The potential of the training increase together with its intensity. The most effective trainings are long-lasting, especially with the practice of the exercises at home.

The context of application results to be irrelevant. Treatments are equally effective in anxiety reduction, both for in-group or individual sessions.

Different anxiety questionnaires present different sensitivity to anxiety changing. Trait anxiety reductions were lower than state anxiety, assessed with the two scales of the STAI. Studies that used the BAI obtained higher effect sizes, maybe due to a greater sensitivity of this instrument.

This meta-analysis deals with scores obtained by anxiety questionnaires and cannot be generalized to other aspects, even if anxiety can be considered as a construct related to a lot of human dimensions. For this reason, this work does not speak about general efficacy of relaxation trainings, but it is limited to the anxiety dimensions.

The meta-analytic findings parallel qualitative reviews revealing that relaxation training has potential for the treatment of anxiety in different populations. Further, this meta-analysis extends the existing literature through facilitation of a better understanding of the variability and clinical significance of anxiety improvement subsequent to relaxation.

## Competing interests

The authors declare that they have no competing interests.

## Authors' contributions

GMM contributed to the concept and design, the development of the review protocol, the literature search, the selection of studies, the data extraction and wrote the first draft of the manuscript, as well as contributing to the development of the final version. FP contributed to the concept and design, the development of the review protocol, the literature search, the selection of studies, the quality assessments of the trials, the data extraction, inputting the data to the statistical software, the data analysis and the development of the final draft. GC contributed to the development of the review protocol, the selection of trials, the quality assessments of the trials and the development of the final draft. EM contributed to the interpretation of the results, critically revised and approved the final manuscript. All authors contributed to the interpretation of the results, read and approved the final manuscript.

## Pre-publication history

The pre-publication history for this paper can be accessed here:


